# 82. Not just another hypermobile patient: Noonan syndrome diagnosed in late adulthood in the rheumatology clinic

**DOI:** 10.1093/rap/rky034.045

**Published:** 2018-09-20

**Authors:** Fang En Sin, Hanadi Kazkaz

**Affiliations:** Rheumatology, University College Hospital, London, UNITED KINGDOM

**Introduction:** We present a case of Noonan syndrome, which was diagnosed for the first time in a lady at the age of 50. She was referred to rheumatology for assessment of hypermobility, but was noted to have an unusual facies which triggered genetic testing. This case highlights the importance of comprehensive systems review and thorough clinical examination in patients with hypermobility.

**Case description:** A 50 year-old lady was referred for assessment of a possible hereditary connective tissue disease following a colonoscopy which found excessive looping in her colon. She has known hypermobility with associated biomechanical pain, diagnosed in another rheumatology unit in the past. She worked full time, was single and had no children. She was very active and held a black belt in kickboxing. However in the past five years, she had presented with multiple symptoms, culminating in 40 clinic appointments with eight medical and surgical specialties, physiotherapy and podiatry, as well as several emergency department attendances. Systemic enquiry revealed a multitude of symptoms including widespread musculoskeletal pain, constipation, thickened skin on her soles and easy bruising. Past medical history include convergent squint, narrow angle glaucoma, two corrective surgeries for ptosis aged 5 and 30, dilated pulmonary artery and pulmonary regurgitation and multiple trauma-related fractures. On examination, she was noted to have unusual facial features including ptosis, low set ears, thin eyebrows, thick curly hair and a short neck. She had a normal body mass index and a height of 167cm. She was hypermobile with a Beighton score of 6/9 and marked flexibility of the small joints of the hands. There was a mild scoliosis and an abnormally shaped chest wall. She had low arched feet with ankle pronation on standing and mild lymphoedema. She had high arched palate, extensible skin but no abnormal scars. Large bruises were noted on her legs. The initial clinical impression was that of a possible genetic collagen disorder, including rare forms of Ehlers Danlos syndrome. Genetic screening was arranged, which revealed an SOS1 mutation in keeping with Noonan syndrome.

**Discussion:** Noonan syndrome is an autosomal dominant disorder with a prevalence of 1 in 1000 to 1 in 2500. Its clinical presentation changes with age. The musculoskeletal system can be affected, with features of small joint hypermobility and scoliosis. Our patient has a mild form of Noonan syndrome, which was only diagnosed at the age of 50. Yet, she exhibited typical clinical features of Noonan syndrome, including unusual facial appearance, small joint hypermobility, cardiac malformation, easy bruising, lymphoedema and ophthalmic manifestations. A number of these seemingly unrelated conditions, some of which may appear trivial in isolation, had been managed as single entities by a wide variety of health care professionals. Her unusual facial and musculoskeletal appearance, coupled by the collection of systemic manifestations, led to genetic testing and a unifying diagnosis. For the patient, this diagnosis was extremely important. She had always felt that she looked different, and now she knew why.

**Key Learning Points:** This case highlights the importance of careful and thorough clinical examination in all patients. In this case, the detection of subtle but unusual physical appearance led to a suspicion and eventually a diagnosis of a genetic disorder. Non-inflammatory, biomechanical musculoskeletal symptoms form a large proportion of new patient caseload in the general rheumatology clinic. Hypermobility, with or without associated musculoskeletal pain, is also increasingly recognised, reflected in increased referrals for its assessment to Rheumatology clinics. It is important for rheumatologist to be aware of the fact that hypermobility can be a feature of genetic disorders other than the commonly known Ehlers Danlos syndrome and Marfan syndrome. Having an open mind, and taking into account patients’ symptoms which may not immediately appear relevant to the problem at hand can be helpful in joining the dots. Having a physician who is able to look at the whole picture, and provide a unifying explanation for their collection of ill-health, can be an extremely positive experience for the patient.

**Disclosure: F. Sin:** None. **H. Kazkaz:** None.

